# Genetically-predicted placental gene expression is associated with birthweight and adult body mass index

**DOI:** 10.1038/s41598-022-26572-6

**Published:** 2023-01-06

**Authors:** Elizabeth A. Jasper, Jacklyn N. Hellwege, Jacqueline A. Piekos, Sarah H. Jones, Katherine E. Hartmann, Brian Mautz, David M. Aronoff, Todd L. Edwards, Digna R. Velez Edwards

**Affiliations:** 1 Vanderbilt Genetics Institute, Vanderbilt University, Nashville, TN USA; 2 Department of Obstetrics and Gynecology, Vanderbilt University Medical Center, Nashville, TN USA; 3 Department of Biomedical Informatics, Vanderbilt University Medical Center, Nashville, TN USA; 4 Division of Genetic Medicine, Department of Medicine, Vanderbilt University Medical Center, Nashville, TN USA; 5 Vanderbilt Epidemiology Center, Vanderbilt University, Nashville, TN USA; 6 Institute for Medicine and Public Health, Vanderbilt University Medical Center, Nashville, TN USA; 7 Population Analytics, Analytics and Insights, Data Sciences, Janssen Research & Development, Spring House, PA USA; 8 Department of Medicine, Indiana University School of Medicine, Indianapolis, IN USA; 9 Division of Epidemiology, Department of Medicine, Vanderbilt University Medical Center, Nashville, TN USA

**Keywords:** Genetics, Diseases, Risk factors

## Abstract

The placenta is critical to human growth and development and has been implicated in health outcomes. Understanding the mechanisms through which the placenta influences perinatal and later-life outcomes requires further investigation. We evaluated the relationships between birthweight and adult body mass index (BMI) and genetically-predicted gene expression in human placenta. Birthweight genome-wide association summary statistics were obtained from the Early Growth Genetics Consortium (N = 298,142). Adult BMI summary statistics were obtained from the GIANT consortium (N = 681,275). We used S-PrediXcan to evaluate associations between the outcomes and predicted gene expression in placental tissue and, to identify genes where placental expression was exclusively associated with the outcomes, compared to 48 other tissues (GTEx v7). We identified 24 genes where predicted placental expression was significantly associated with birthweight, 15 of which were not associated with birthweight in any other tissue. One of these genes has been previously linked to birthweight. Analyses identified 182 genes where placental expression was associated with adult BMI, 110 were not associated with BMI in any other tissue. Eleven genes that had placental gene expression levels exclusively associated with BMI have been previously associated with BMI. Expression of a single gene, *PAX4*, was associated with both outcomes exclusively in the placenta. Inter-individual variation of gene expression in placental tissue may contribute to observed variation in birthweight and adult BMI, supporting developmental origins hypothesis.

## Introduction

Between 1999 and 2018, the prevalence of adult obesity has increased by almost 12%, with over 40% of Americans over the age of 20 currently being classified as obese^1^. Obesity, commonly assessed using body mass index (BMI), has a significant impact on individuals’ health, with obese individuals at increased risk for numerous diseases and conditions, including all-cause mortality, hypertension, dyslipidemia, Type 2 diabetes, and heart disease^2^. In 2016, the health consequences of obesity were estimated to account for $1.72 trillion dollars in total health care costs in the United States^3^. Though a major public health issue, existing strategies for prevention and intervention in adults have had limited to moderate success, suggesting that the understanding of the mechanisms contributing to the variation in BMI and underlying development of obesity is incomplete^4^. Given that the majority of obesity interventions are based on modifying known risk factors, further research investigating determinants of obesity could provide key insights into etiology and additional actionable targets.

BMI and obesity are complex traits with etiologies that are multifactorial, involving genetic and environmental factors as well as the interaction between these factors^5^. Several factors during the preconception, fetal, and infant periods possess substantial impact on adult obesity, such as gestational weight gain, exposure to diabetes in utero, and genetic variation. Weight, BMI, and obesity status earlier in life are significant predictors risk factors for adult BMI and obesity, as BMI tends to track from early childhood and adolescence into adulthood^6,7^. Specific growth trajectories in infancy and early childhood also increase the risk of obesity in later life^8–13^. Birthweight is also consistently associated adult BMI and obesity throughout the life course^14^. Most observational studies linking birthweight with later anthropomorphic measures have found a linear relationship between birthweight and adult BMI, overweight and obesity status of offspring. Recent investigations suggest those born at low birthweight (< 2500 g) have a decreased risk of later overweight status (odds ratio (OR): 0.73, 95% confidence interval: 0.63–0.84) while overweight adults are 1.60 (95% confidence interval: 1.45–1.77) times more likely to have high birthweight (> 4000 g) compared to individuals who are not obese in adulthood^15^.

Though a strong relationship between birthweight and adult BMI or obesity has been consistently found in observational epidemiologic research, the causal mechanisms leading to the variation in birthweight and later-life BMI, as well as the etiologic mechanisms underlying their association remains unclear. New approaches are necessary because classical, observation epidemiologic research has not provided a complete explanation for these outcomes. With growing evidence that both have origins in the perinatal period, the Developmental Origins of Health and Disease (DOHaD) theory, a model for disease causation that states that an individual’s health throughout their life course is significantly influenced by exposures during pregnancy^16,17^, may provide a framework that can guide research on causal mechanisms. The theory suggests the environment in utero and early postnatal life programs individuals’ characteristics and contributes to disease(s) later in life. Programming in this context largely occurs through epigenetics, resulting in changes in gene expression. Thus, this framework moves beyond environmental and genetic risk factors for these outcomes and steers research toward potential biologic and molecular mechanisms.

Under the DOHaD framework, the placenta is an important biological conduit to mediate genomic and non-genomic transmission of risk for noncommunicable diseases and traits like birthweight and BMI. As the physical and functional connection between a mother and developing fetus, the placenta is critical to fetal growth and development^18^. The genetic regulation of placental gene expression has recently been described through expression quantitative trait loci (eQTL) studies^19^, which have identified associations with childhood phenotypes^20^. Because germline DNA is unvarying from conception, gene expression in placental tissue can be predicted in newborns and adult individuals using genome-wide association (GWAS) data.

The objective of this study was to dissect the relationship between adult BMI and genetically-predicted gene expression (hereby referred to only as gene expression) in the placenta, with the underlying premise that alterations in placental-specific gene expression would be associated with individuals’ BMI later in life. Given that birthweight is consistently associated with later life BMI and the placenta plays a key role in fetal growth, we also evaluated the association between gene expression and birthweight. As there is some overlap between known birthweight and adult BMI loci^21,22^, we compared the results from the birthweight analysis to the results of the BMI analysis. Since the existence of the placenta is considerably closer to the birthweight phenotype, we hypothesized that there would be substantial differences in the genes where expression in the placenta associates with birthweight versus adult BMI.

## Results

### Birthweight z-score gene expression results

**Figure 1:**
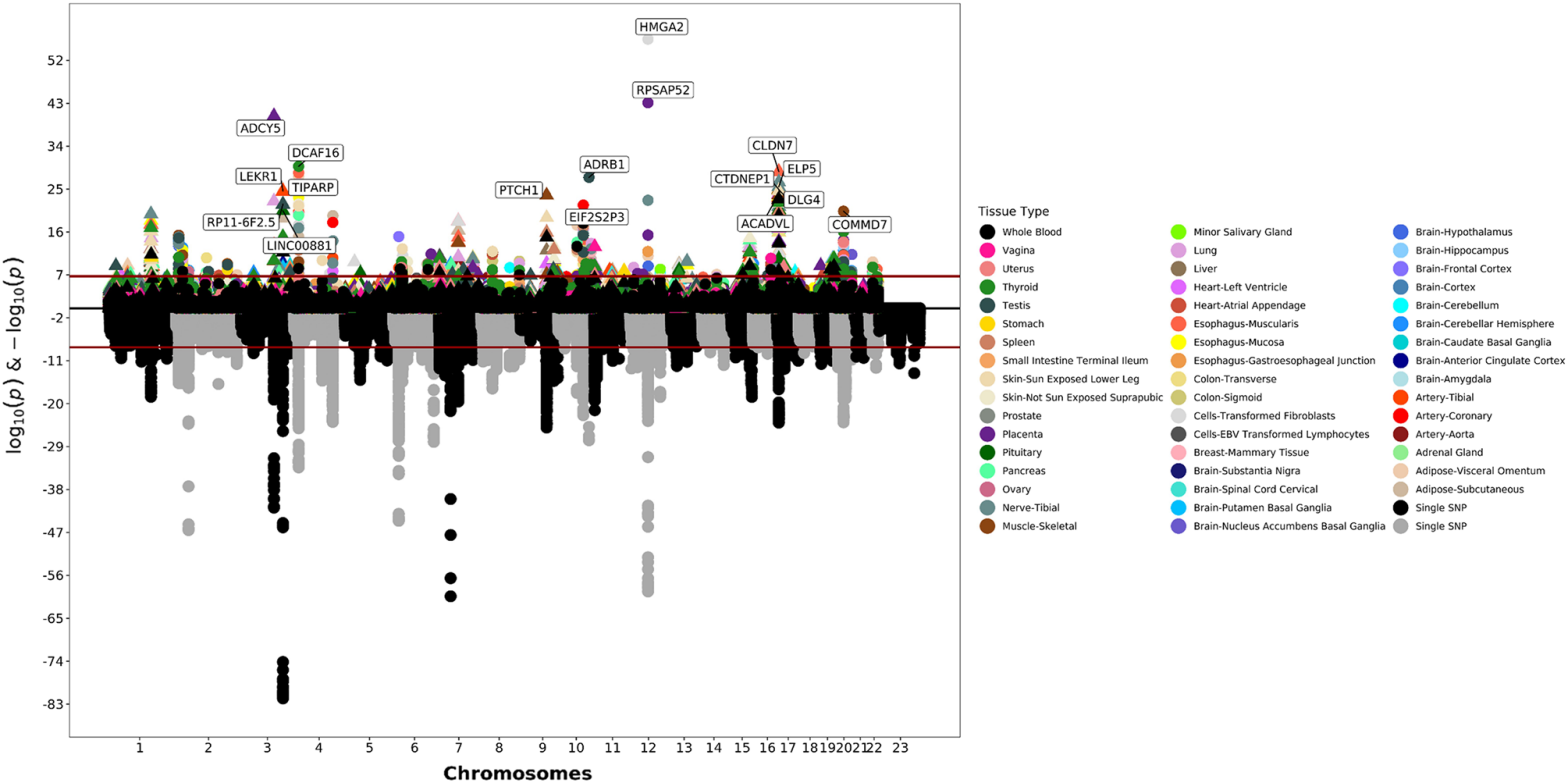
Birthweight Miami Plot. The bottom of the graphic is a Manhattan plot which displays significant SNPs from GWAS. The top of the graphic is the results from S-PrediXcan, with symbols now representing entire genes and their genetically determined expression levels. The x-axis are chromosomes. The y-axis is log and negative log *p*-values from the GWAS and S-PrediXcan analyses. Colors correspond to specific tissues.

Across the 49 tissues, we tested the association between 263,683 genes’ expression and birthweight z-score (Fig. 1; Supplementary Table S1). More than 40,000 (15.19%) of the tested expression-birthweight z-score associations had suggestive significance at the 0.05 level; however, 804 (0.30%) gene expression results were significant after Bonferroni correction. The most significant result was with predicted *HMGA2* expression in transformed fibroblast cells (Supplementary Table S2). Increased *HMGA2* expression in this tissue was associated with increasing birthweight z-score (effect size: 0.35 standard deviations [SD] of birthweight per SD of *HMGA2* expression, *p*-value: 3.58 × 10^−57^). Increased expression of this gene was also highly significant in the placenta (effect size: 0.04 SD of birthweight per SD of *HMGA2* expression, *p*-value: 4.32 × 10^−16^). The second most significant result was a positive association between expression of *RPSAP52* and birthweight z-score in the placenta (effect size: 0.11 SD of birthweight per SD of *RPSAP52* expression, *p*-value: 7.49 × 10^−44^). Expression of *ADCY5* was also associated with birthweight z-score in the placenta (effect size: -0.09 SD of birthweight per SD of *ADCY5* expression, *p*-value: 4.49 × 10^−41^). Both *HGMA2*^23–26^ and *ADCY5*^23–27^ have been previously implicated in GWAS studies for birthweight. *RPSAP52*, which was only significantly associated with birthweight in placental tissue, has not been previously associated with birthweight before but has been associated with Type 2 diabetes^28^. Within the 804 significant associations, there were 148 genes where expression was only significant in a single tissue (Supplementary Table S3).

Among the statistically significant gene expression association results, there were 24 genes (Supplementary Table S4) for which placental expression was associated with birthweight z-score. Fifteen of these 24 associations were specific to the placenta, as they were not significantly associated with birthweight z-score in any other tissue (Table 1). Six of the genes where expression was significantly associated with birthweight z-score in the placenta have been previously associated with birthweight: *ADCY5*^23^, *HMGA2*^23^, *TNFSF12*^23^ *PLEKHA1*^24,26^, *FES*^26^, and *SLC38A1*^23^. *SLC38A1* was the only one of these genes where expression was only associated with birthweight in placental tissue (Table 1).

**Table 1:** Genes where expression was significant exclusively in the placenta for birthweight.

Gene	Effect size (Standard deviations [SD] of birthweight per SD of gene expression)	Standard error	*p*-value
*RPSAP52*	0.115	0.0082	7.49 × 10^−44^
*RP11-63E9.1*	0.046	0.0067	4.00 × 10^−12^
*KRT18P67*	− 0.034	0.0052	5.07 × 10^−11^
*VARS*	− 0.035	0.0055	4.78 × 10^−10^
*KDM4B*	− 0.044	0.0072	1.04 × 10^−9^
*SPATA8*	0.028	0.0047	2.86 × 10^−9^
*PAX4*	0.023	0.0038	2.89 × 10^−9^
*AC051649.6*	− 0.031	0.0055	2.36 × 10^−8^
*RP11-351I24.3*	0.020	0.0037	2.82 × 10^−8^
*MDC1*	− 0.034	0.0063	4.50 × 10^−8^
*FGFR1OP2*	− 0.034	0.0062	4.62 × 10^−8^
*SLC38A1*	0.039	0.0073	6.61 × 10^−8^
*GNG11*	0.046	0.0086	8.02 × 10^−8^
*ZNF425*	− 0.049	0.0092	1.00 × 10^−7^
*E2F3-IT1*	0.033	0.0064	1.67 × 10^−7^

### Adult BMI gene expression results

**Figure 2:**
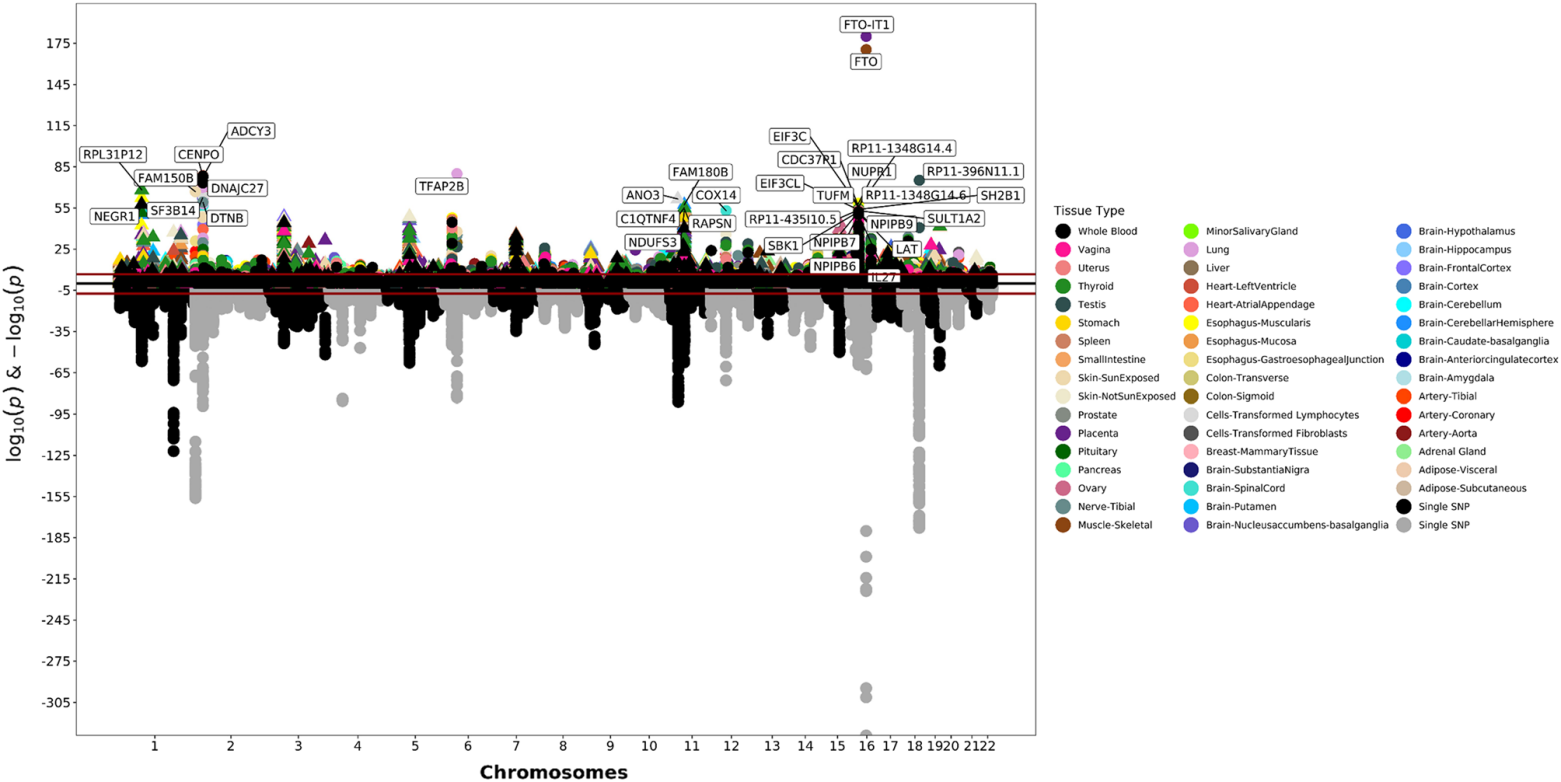
Adult Body Mass Index Miami Plot. The bottom of the graphic is a Manhattan plot which displays significant SNPs from GWAS. The top of the graphic is the results from S-PrediXcan, with symbols now representing entire genes and their genetically determined expression levels. The x-axis are chromosomes. The y-axis is log and negative log p-values from the GWAS and S-PrediXcan analyses. Colors correspond to specific tissues.

The adult BMI analysis consisted of 258,869 association tests (Fig. 2; Supplementary Table S5). More than 30% (78,679 associations) of the tested expression-BMI associations had suggestive significance at the 0.05 level, 8,834 (11.23%) of which were significant after correction for multiple testing (Supplementary Table S6). The most significant gene was *FTO-IT1*, with increasing expression in the placenta associated with increasing BMI (effect size: 0.12 kg/m^2^ per SD of *FTO-IT1* expression, *p*-value: 8.20 × 10^−181^). The second most significant result was a positive association between increasing predicted expression of *FTO* and BMI in the skeletal muscle tissue (effect size: 0.20 kg/m^2^ per SD of *FTO* expression, *p*-value: 4.38 × 10^−171^). Previous GWAS studies have tied variants in *FTO* to BMI in infancy, childhood, and adulthood. The association between *FTO* variants and BMI is one of the strongest associations documented and has been widely replicated in numerous populations^29–33^. One thousand one hundred and forty-eight genes’ expression was only significant in a single tissue (Supplementary Table S7).

Of the significant associations, the placental expression of 182 genes was linked to BMI (Supplementary Table S8). When comparing against predicted expression models from 48 other tissues from GTEx, 110 of the significant genes in the placenta were unique and not associated with BMI when analyzed in any other tissue (Table 2). *FTO-IT1*, the most significant gene of all tested associations, was one of the genes where placental expression was exclusively associated with adult BMI. Many significant genes have been previously tied to BMI through their association with regulatory, intergenic, or genic variants. Within these genes, 50 were significant in placental tissue, with 11 genes significantly associated with BMI exclusively in placental tissue. These 11 genes are: *TOMM40*^32,34–38^, *LINC00971*^37^, *RBBP6*^35–37,39–41^, *FAM169A*^37^, *RPL21P119*^40^, *ZZZ3*^36,38,42^, *CREB1*^41^, *GRIA1*^37,40^, *POU6F2-AS1*^40^, *LIMD1*^37^, and *ZFYVE1*^37^ (Table 2; Supplementary Table S8).

**Table 2:** Genes where expression was significant exclusively in the placenta for adult BMI.

Gene	Effect size (kg/m^2^ per SD of gene expression)	Standard error	*p*-value
*FTO-IT1*	0.116	0.004	8.20 × 10^–181^
*RP11-537I16.2*	0.077	0.007	2.80 × 10^–32^
*TOMM40*	0.041	0.004	2.60 × 10^–25^
*MIR1915*	0.062	0.006	4.00 × 10^–25^
*RNA5SP20*	0.053	0.005	2.40 × 10^–22^
*MIR1301*	0.052	0.006	1.40 × 10^–20^
*RP11-115J16.1*	0.008	0.001	1.44 × 10^–20^
*RP5-849H19.2*	− 0.045	0.005	1.80 × 10^–18^
*Y_RNA* (ENSG00000206717)	− 0.045	0.005	2.90 × 10^–18^
*RP11-10A14.3*	− 0.048	0.006	5.50 × 10^–18^
*ARHGEF26-AS1*	0.031	0.004	3.80 × 10^–17^
*PAM16*	0.046	0.006	9.40 × 10^–17^
*HSPE1P25*	0.047	0.006	1.30 × 10^–16^
*RP11-120B7.1*	0.026	0.003	8.79 × 10^–16^
*RP11-96D1.6*	0.035	0.004	5.50 × 10^–15^
*RP11-501C14.6*	− 0.036	0.005	1.80 × 10^–14^
*LINC00971*	0.044	0.006	5.90 × 10^–14^
*RP11-455F5.4*	0.043	0.006	6.00 × 10^–14^
*FTH1P12*	0.022	0.003	1.45 × 10^–13^
*RP11-977G19.5*	0.041	0.006	2.20 × 10^–13^
*PLB1*	0.040	0.006	3.80 × 10^–13^
*RP11-61I13.3*	0.035	0.005	5.80 × 10^–13^
*ZNF521*	0.026	0.004	5.93 × 10^–13^
*RNU6-1309P*	0.038	0.005	6.00 × 10^–13^
*ENTHD2*	− 0.042	0.006	9.60 × 10^–13^
*AC012358.7*	0.029	0.004	1.40 × 10^–12^
*FKBP1AP2*	− 0.036	0.005	2.00 × 10^–12^
*RPUSD1*	0.035	0.005	2.20 × 10^–12^
*RP11-117L6.1*	− 0.014	0.002	2.70 × 10^–12^
*RNU4-78P*	0.033	0.005	3.10 × 10^–12^
*AP000859.4*	− 0.032	0.005	3.40 × 10^–12^
*Y_RNA* (ENSG00000200544)	− 0.033	0.005	3.50 × 10^–12^
*RBBP6*	− 0.026	0.004	4.38 × 10^–12^
*FAM169A*	0.030	0.004	5.30 × 10^–12^
*GRAMD2*	− 0.017	0.002	6.31 × 10^–12^
*RPL27P12*	− 0.037	0.005	7.60 × 10^–12^
*RPL21P119*	0.019	0.003	1.16 × 10^–11^
*RGPD1*	− 0.037	0.006	1.70 × 10^–11^
*HOGA1*	0.019	0.003	2.11 × 10^–11^
*ZNF597*	− 0.034	0.005	2.90 × 10^–11^
*IQCD*	0.033	0.005	3.00 × 10^–11^
*ZZZ3*	0.036	0.006	4.60 × 10^–11^
*CCM2*	0.034	0.005	4.90 × 10^–11^
*BGLAP*	− 0.034	0.005	7.60 × 10^–11^
*LFNG*	− 0.026	0.004	8.20 × 10^–11^
*REG1B*	0.027	0.004	8.80 × 10^–11^
*Y_RNA* (ENSG00000223187)	− 0.020	0.003	9.75 × 10^–11^
*RNA5SP290*	− 0.029	0.004	1.00 × 10^–10^
*ARPC3P1*	− 0.031	0.005	1.00 × 10^–10^
*GBX2*	− 0.029	0.005	1.40 × 10^–10^
*CREB1*	0.034	0.005	2.30 × 10^–10^
*HTR3C*	− 0.030	0.005	2.70 × 10^–10^
*POU6F2-AS2*	0.038	0.006	2.80 × 10^–10^
*CTC-484P3.3*	0.019	0.003	3.00 × 10^–10^
*GRIA1*	0.029	0.005	3.05 × 10^–10^
*RP11-172E9.2*	− 0.032	0.005	3.70 × 10^–10^
*AC092159.3*	0.020	0.003	4.04 × 10^–10^
*AC099668.5*	0.036	0.006	5.10 × 10^–10^
*LSM7*	0.022	0.004	5.61 × 10^–10^
*RP11-472I20.1*	− 0.031	0.005	5.70 × 10^–10^
*CTD-2244F11.2*	0.022	0.004	5.86 × 10^–10^
*RP1-200K18.1*	0.016	0.003	1.54 × 10^–9^
*RETNLB*	0.026	0.004	1.60 × 10^–9^
*BMPR1A*	0.019	0.003	1.62 × 10^–9^
*RP11-87C12.2*	− 0.025	0.004	1.80 × 10^–9^
*CAMK1G*	− 0.035	0.006	2.10 × 10^–9^
*RP11-165J3.6*	− 0.021	0.003	2.28 × 10^–9^
*RP11-328C8.2*	0.016	0.003	3.40 × 10^–9^
*RP5-837I24.4*	0.018	0.003	4.95 × 10^–9^
*BRWD1-IT2*	− 0.026	0.005	5.90 × 10^–9^
*RP1-5O6.5*	− 0.035	0.006	6.30 × 10^–9^
*RP11-270C12.3*	0.016	0.003	7.76 × 10^–9^
*RP11-212F11.1*	− 0.019	0.003	1.02 × 10^–8^
*RP11-448P19.1*	− 0.020	0.004	1.41 × 10^–8^
*MMP19*	− 0.029	0.005	1.60 × 10^–8^
*RN7SL814P*	− 0.033	0.006	1.70 × 10^–8^
*SIGLEC6*	− 0.022	0.004	1.75 × 10^–8^
*ERP44*	0.006	0.001	1.88 × 10^–8^
*POU6F2-AS1*	− 0.035	0.006	1.90 × 10^–8^
*ABHD1*	0.038	0.007	2.40 × 10^–8^
*RP11-166D19.1*	0.014	0.003	2.48 × 10^–8^
*AC113607.2*	0.019	0.003	2.58 × 10^–8^
*BUD13*	0.029	0.005	2.60 × 10^–8^
*SEC22C*	0.036	0.007	2.80 × 10^–8^
*LIMD1*	− 0.018	0.003	3.06 × 10^–8^
*RP11-252I13.2*	− 0.032	0.006	4.00 × 10^–8^
*UBN2*	− 0.029	0.005	4.00 × 10^–8^
*SRGAP3*	0.030	0.006	4.00 × 10^–8^
*PCNXL3*	− 0.027	0.005	4.10 × 10^–8^
*XXbac-B444P24.8*	0.021	0.004	4.40 × 10^–8^
*RP11-2A1.1*	− 0.013	0.002	4.62 × 10^–8^
*RP11-109N23.1*	0.031	0.006	5.40 × 10^–8^
*MIR382*	0.044	0.008	5.50 × 10^–8^
*SLC22A7*	0.025	0.005	6.80 × 10^–8^
*PAX4*	0.029	0.005	7.00 × 10^–8^
*NPM1P38*	− 0.029	0.005	7.60 × 10^–8^
*SNORA62*	− 0.031	0.006	9.60 × 10^–8^
*FAM160A1*	0.024	0.004	1.10 × 10^–7^
*AC092159.2*	− 0.026	0.005	1.10 × 10^–7^
*C11orf54*	0.027	0.005	1.10 × 10^–7^
*RP11-421P23.1*	0.017	0.003	1.18 × 10^–7^
*CHPF*	0.031	0.006	1.20 × 10^–7^
*RN7SKP200*	0.022	0.004	1.30 × 10^–7^
*POLR1C*	− 0.021	0.004	1.42 × 10^–7^
*AC113607.3*	0.021	0.004	1.69 × 10^–7^
*C18orf54*	0.014	0.003	1.73 × 10^–7^
*RP11-211C9.1*	− 0.017	0.003	1.76 × 10^–7^
*CTD-2196E14.6*	0.028	0.005	1.80 × 10^–7^
*ZFYVE1*	− 0.028	0.005	1.80 × 10^–7^
*PRKAG3*	− 0.053	0.010	1.90 × 10^–7^

### Overlapping results across adult BMI and birthweight for the placenta

A single gene was significantly associated with both birthweight and adult BMI in placental tissue: *PAX4*. For both outcomes, *PAX4* was only significant in the placental tissue. The direction of effect was consistent between both phenotypes (birthweight effect size: 0.02 SD of birthweight per SD of *PAX4* expression, *p*-value: 2.89 × 10^−9^; BMI effect size: 0.03 kg/m^2^ per SD of *PAX4* expression, *p*-value: 7.00 × 10^–8^) but it has not been tied to either outcome previously.

### Gene enrichment

Evaluation of gene set enrichment among genes associated with birthweight in the placenta identified a significantly enriched reactome pathway: transmission across chemical synapses, which featured four birthweight-placental gene expression genes among the 269 involved in the pathway (*p*-value_adj_ = 0.027) (Supplementary Fig. S1). There were no GO biological processes, GO cellular components, or molecular functions that were enriched. Evaluation of gene set enrichment among genes associated with adult BMI in the placenta did not identify enriched GO biologic processes, GO cellular components, or reactome pathways.

## Discussion

This study evaluated associations between gene expression and body weight traits, in the context of large-scale GWAS. We observed evidence that specific genes exert effects on these traits through expression levels in placenta and did not observe evidence of effects from these genes from adult tissues. This suggests that the process leading to birthweight and adult body composition that begins with genetic factors has intermediate steps that occur as early as the placenta, and that do not have effects in other tissues. This supports the DOHaD hypothesis, and these studies that identify genes and tissue context may provide biomarkers or drug targets for adverse outcomes.

Expression of 804 genes was significantly associated with birthweight, while 8,834 genes’ predicted expression was associated with adult BMI. Predicted expression of 24 and 182 of these genes was significantly associated with birthweight and BMI in placenta tissue, respectively. However, placental expression of only 15 of the genes associated with birthweight and 110 of those associated with BMI were significantly and exclusively associated with these outcomes. Many of the genes where expression levels were only linked to the outcome in the placenta have not been previously associated with birthweight or BMI, as single nucleotide polymorphisms (SNPs) in only one (*SLC38A1*)^23^ and 11 (*TOMM40*^32,34–38^, *LINC00971*^37^, *RBBP6*^35–37,39–41^, *FAM169A*^37^, *RPL21P119*^40^, *ZZZ3*^36,38,42^, *CREB1*^41^, *GRIA1*^37,40^, *POU6F2-AS1*^40^, *LIMD1*^37^, *ZFYVE1*^37^) of these genes have been associated with birthweight and adult BMI in previous GWAS, respectively. Thus, our study replicates the association between these previously reported genes and birthweight and BMI, providing a tissue-specific mechanism of action. Importantly, this study identified numerous novel associations, tying previously unknown genes to these outcomes through their expression in various tissues.

*ADCY5* was significantly associated with birthweight in the placenta. It has been consistently associated with birthweight and Type II diabetes^27,43^. Though the causal mechanisms are unknown, a previous EGG Consortium study noted its pleiotropic effects on glucose regulation and Type II diabetes in adulthood. One potential mechanism explaining the association between birthweight, diabetes, and the gene is the fetal insulin hypothesis. Under this hypothesis, *ADCY5* could impact insulin secretion and its risk allele would have a direct effect on fetal growth via reduced insulin secretion^27,44^. Of the genes associated only with birthweight in the placenta, *SLC38A1* was the only one previously associated with birthweight^23^. The protein encoded by this gene is an amino acid transporter and plays critical roles in the uptake of nutrients, energy production, and chemical metabolism. The other 14 genes with expression levels only associated with the outcome in the placenta have not been previously tied to birthweight. The only enrichment among the genes associated with birthweight in the placenta was for a reactome pathway (transmission across chemical synapses). No known biological process or molecular function was over- or under-represented in this collection of genes. However, variants in these genes have been associated with related phenotypes. Several variants nearby implicated genes are associated with metabolic and hormonal traits, such as fasting blood glucose (*VARS*)^45^, triglyceride measurement (*KDM4B*)^46^, and 17-hydroxyprogesterone (*MDC1*)^47^, and may represent diverse molecular pathways involved in regulation of birthweight. Polymorphisms in these genes have also been tied to Type II diabetes (*PAX4*)^48^, height (*KDM4B*)^40^, and growth retardation and diabetic complications in mammalian knockout models (*PAX4*)^49^. Furthermore, two genes which were not previously reported as associated with birthweight (*MDC1*, *PAX4*), as well as *SLC38A1*, exist in topologically associated domains with SNPs that were previously associated with birthweight^23^. These observations from Warrington et al. support the validity of our findings that genetically predicted gene expression of these genes is associated with inter-individual variation in birth weight.

We found a larger number of genes where placental expression, specifically, was associated with BMI. Again, the biological processes and molecular functions of the genes varied widely. Notable findings include the negative association between *SIGLEC6* expression and BMI (*p*-value: 1.75 × 10^−8^). This gene is expressed nearly exclusively in the placenta and nearby associations with BMI have previously been attributed to other genes. However, there is a clear potential causal mechanism linking *SIGLEC6* and BMI. The gene encodes a transmembrane receptor that binds sialyl-TN glycans and leptin, the latter of which is a hormone predominantly made by adipose cells and functions in energy homeostasis, neuroendocrine function, and metabolism. *SIGLEC6* is also involved in sialic acid biology and its increased expression has been found in placentas from pregnancies complicated by preterm preeclampsia^50^. The most significant gene of all tested associations was *FTO-IT1*. Its expression was exclusively associated with adult BMI in the placenta with increased expression associated with an increase BMI. Though the exact physiological function of the gene is unknown, as a long noncoding ribonucleic acid it likely functions in transcription regulation, potentially of the FTO gene which has a well-known association with weight and BMI and is hypothesized to play a role in regulation of appetite^51^.

Gene expression of *PAX4* was the only commonality between the birthweight and BMI analyses. For both birthweight and BMI, this gene’s expression was significantly tied to the outcomes exclusively in the placenta. *PAX4*, a member of the paired-box family of transcription factors, plays a critical role in fetal development, particularly in the differentiation and development of pancreatic islet beta cells. This gene has not been previously tied to birthweight or BMI but has been linked to Type II diabetes, a phenotype closely linked to increasing BMI, as well as energy storage, metabolism, and homeostasis.

In our study, birthweight and adult BMI had limited similarities in gene expression patterns in the placenta. This could be due to the timing of the phenotypes in relation to the existence of the placenta. Differences may occur due to biological proximity of the placenta to birthweight compared to adult BMI. The small overlap between the two phenotypes’ genes where expression was significantly associated with the outcome exclusively in placental tissue is in line with other studies of weight and BMI, which have found differing genetic factors influence these outcomes at different periods in life^52,53^. Another likely contributor to the differences in placental gene expression associations with birthweight and adult BMI is their phenotype definitions. The neonatal outcome of birthweight is the sum of both fat-free mass and fat mass^54^. It does not discriminate between these two masses. Changes in weight could represent alterations in fat, muscle, fluids, bone, or combinations of these components^54^. Thus, body weight, though often used to assess nutritional and health status, is unable to quantify body composition and is a poor indicator of obesity. In comparison, BMI is a measure of weight adjusted for height^55^. It incorporates components of both body structure (e.g., skeletal mass, limb length, etc.) and body composition (e.g., fat mass, etc.)^56^. Differences in placental expression patterns may be due to BMI accounting for height; whereas, the neonatal phenotype does not account for body shape. Regardless of the small overlap in significant placental gene expression, these results highlight the importance of the gestational period in defining disease risk^57^.

As this study utilized genetically-predicted gene expression, expression level estimates did not account for components of expression attributable to environment and other factors. However, our approach of estimating genetically-predicted gene expression and its associations with outcomes is not subject to confounding due to trait- or outcome-altered expression. Direct measurement of expression levels in such large samples would also require prohibitive resources. Thus, the use of S-PrediXcan offers the advantage of estimation of transcriptome measurement and its association with the outcomes without requiring actual transcript measurements. S-PrediXcan also takes GWAS summary statistics as input, allowing us to use publicly available datasets, leverage larger populations for both birthweight and BMI analyses, and achieve higher power. This study may have limited generalizability as both populations contained only those of European ancestry individuals. Future analyses should replicate this approach in more diverse, trans-ancestry populations as the data becomes available. Since birthweight and BMI have been linked to numerous outcomes, including gestational age, childhood BMI, and obesity, future studies should compare these results to results from S-PrediXcan analyses of related phenotypes. Studies directly measuring expression levels in the placenta and relating them to birthweight and BMI are also necessary and would aid in furthering the understand of environmental and trait-related effects on transcription.

We were unable to investigate potential impact or confounding effects caused by correlation between maternal and fetal genotypes. However, our results rely entirely on fetal genotypes and the placenta model is built using expression data collected exclusively from the neonatal side of the placenta. Therefore, it is unlikely that the results are due to this path of confounding. Future studies should aim to collect maternal data to account for correlation between maternal and fetal genotypes and compare results between them. Sample sizes for the placenta and 48 GTEx tissues varied (Supplementary Table S9). Variable sample size may have impacted discovery. In cases of drastically different sample sizes between the placenta and non-placental tissue, caution should be taken when comparing and interpreting results due to the potential differences in power. In these cases, further comparison based on the relative effect size might be more appropriate.

This study found hundreds of genes with expression associated with birthweight and adult BMI, several of which were only significantly associated in placental tissue. Many of the genes where expression was exclusively associated with the outcomes in the placenta have not been previously linked to birthweight or BMI. However, there were several genes, where expression was associated with the outcomes in only the placenta, which contain polymorphisms previously implicated in birthweight and BMI. This furthers the mechanistic understanding of the discoveries of previous studies as this analysis tested the molecular mechanisms through which genetic variation affects outcomes. Interestingly, limited overlap in the genes that were only associated with the outcomes in the placenta was observed between birthweight and adult BMI. This is in line with previous studies of weight and BMI, which have suggested that different genetic factors influence these outcomes at different periods in life. In conclusion, inter-individual variation of gene expression in placental tissue may contribute to the observed variation observed in short-term outcomes, like birthweight, as well as long-term outcomes, like adult BMI. Results of this study further support a developmental origin of both birthweight and adult BMI, with placental gene expression providing a possible mechanistic link between exposures in utero and later life health.

## Methods

### Birthweight summary statistics

Birthweight summary statistics were obtained from the most recent Early Growth Genetics Consortium (EGG) GWAS^24^. The EGG Consortium birthweight summary statistics were downloaded at www.egg-consortium.org. The EGG summary statistics were obtained from a genome-wide meta-analysis of birthweight z-score, which combined data from the EGG Consortium and the UK Biobank^23,58^. The summary statistics contained up to 298,142 European ancestry participants.

### Body mass index summary statistics

Adult BMI GWAS summary statistics were obtained from the most recent Genetic Investigation of ANthropometric Traits (GIANT) consortium publication^22^. Up to 681,275 individuals, obtained from the UK Biobank and 114 other GWAS studies, were included in the analyses of roughly 2.4 million SNPs.

### Placental gene expression source data

Placental gene expression and eQTLs were evaluated and computed using published gene expression data from the Rhode Island Child Health Study^19^. Gene expression data on 150 samples were derived from placenta tissue excluding maternal decidua and processed using whole transcriptome RNAseq. Whole genome genotyping (Illumina MEGAex Array, Illumina Inc., San Diego, CA) was used for generating eQTLs, which have been previously published^19^.

### Construction of placental gene expression models

The eQTL summary statistics were processed into genetically predicted expression models. Total eQTLs were filtered within each gene for false discovery rate (FDR)-adjusted p-value less than 0.1 and linkage disequilibrium (LD) clumping was performed (0.1 r^2^ and 250 kilobase window). To retain only those genes with substantial genetic regulation in the placenta, the variance explained by each eQTL SNP was calculated as 2pqβ^2^, where β is the effect size for eQTL association from the original summary statistics file, *p* is the frequency of allele 1, and q is the frequency of allele 2. The sum of SNP variances was computed for each gene and genes were ordered by expression variance explained. Genes with variance of greater than two and less than 0.01 were excluded from the final prediction models. Final models utilized 25,885 genetic variants associated with expression of 15,154 genes.

### Genetically-predicted gene expression analyses

Gene expression was estimated in placental tissue using birthweight and adult BMI summary GWAS statistics separately with S-PrediXcan. S-PrediXcan calculates effects and tests for association between outcomes and the genetically determined component of expression for genes in each tissue using SNP-level association and eQTL summary statistics^59^. To identify genes where expression levels are only associated with the outcomes in the placenta, S-PrediXcan was also used to obtain gene expression estimates in 48 other tissues, for both phenotypes, using existing models from the Genotype Tissue Expression (GTEx v7) project (predictdb.org). GTEx and prediction models based on the data from it utilized samples from non-diseased tissues collected from both male and female donors from a variety of races and ages. Sample sizes varied based on tissue and ranged from 70 to over 400 for version 7. Gene associations with p-values less than 0.05 were considered nominally significant. All effect sizes are presented with regard to increasing predicted gene expression. Genes significantly associated with the outcomes in the placenta were compared to results in the GWAS Catalog. Genes reported and mapped using the GWAS Catalog rules for each outcome were considered and discussed in results as previously implicated in GWAS studies. All results are based on publicly available summary statistics and do not constitute human subjects research.

### Functional annotation

Significant genes from the birthweight and BMI analysis were compared. The web-based annotation tool Functional Mapping and Annotation of Genome-Wide Association Studies (FUMA) gene2func was used to test for their enrichment in cell types and pre-defined biological pathways for all significant associations in the placenta^60,61^.

## Supplementary Information

Supplementary Information 1.

Supplementary Information 2.

Supplementary Information 3.

## Data Availability

Publicly available data analyzed during this study are included in published studies from the Early Growth Genetics (EGG) Consortium^23^ and the Genetic Investigation of ANthropometric Traits (GIANT) consortium^22^. Any additional information and data are available upon reasonable request. The data and materials can be shared by Dr. Digna R. Velez Edwards (Digna.r.velez.edwards@vumc.org) upon reasonable request.
