# Supplementary material for: Genetically-predicted placental gene expression is associated with birthweight and adult body mass index
Source: Sci Rep. 2023 Jan 6;13:322. doi: 10.1038/s41598-022-26572-6 (PMC9822919; doi:10.1038/s41598-022-26572-6)
Supplement: Supplementary file 1 — Supplementary Information 1. [file 41598_2022_26572_MOESM1_ESM.docx]

**Supplementary Table and Figure Legends**

Supplementary Table S1: Results for All Genetically Predicted Gene Expression and Birthweight Association Analyses

Supplementary Table S2: Significant Associations with Birthweight Across All Tissues

Supplementary Table S3: Genes Where Expression Was Significantly Associated With Birthweight in a Single Tissue

Supplementary Table S4: Genes Where Expression Was Significantly Associated with Birthweight in Placental Tissue

Supplementary Table S5: Results for All Genetically Predicted Gene Expression and Adult Body Mass Index Association Analyses

Supplementary Table S6: Significant Associations with Adult BMI Across All Tissues

Supplementary Table S7: Genes Where Expression Was Significantly Associated With Adult BMI in a Single Tissue

Supplementary Table S8: Genes Where Expression Was Significantly Associated with Adult BMI in Placental Tissue

Supplementary Table S9: Sample Size for GTEx Tissues

Supplementary Figure S1: Enriched Reactome Pathway with Placental Genes Associated with Birthweight
