# Supplementary figures and images for: Genetically-predicted placental gene expression is associated with birthweight and adult body mass index
Source: Sci Rep. 2023 Jan 6;13:322. doi: 10.1038/s41598-022-26572-6 (PMC9822919; doi:10.1038/s41598-022-26572-6)

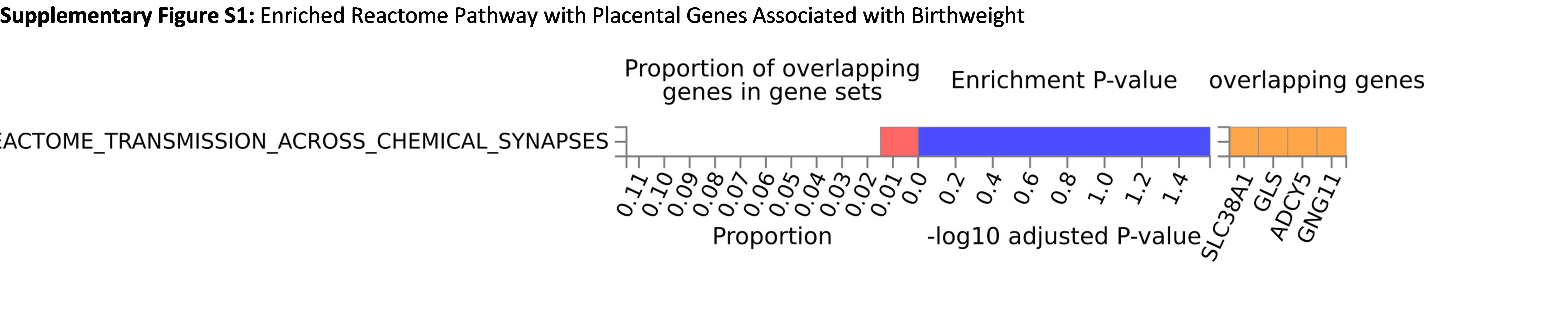

Supplement: Supplementary file 3 — Supplementary Information 3. [file 41598_2022_26572_MOESM3_ESM.jpg]
